# Antioxidant Defence, Oxidative Stress and Oxidative Damage in Saliva, Plasma and Erythrocytes of Dementia Patients. Can Salivary AGE be a Marker of Dementia?

**DOI:** 10.3390/ijms18102205

**Published:** 2017-10-20

**Authors:** Magdalena Choromańska, Anna Klimiuk, Paula Kostecka-Sochoń, Karolina Wilczyńska, Mikołaj Kwiatkowski, Natalia Okuniewska, Napoleon Waszkiewicz, Anna Zalewska, Mateusz Maciejczyk

**Affiliations:** 1 Department of Restorative Dentistry, Medical University of Bialystok, 15-276 Bialystok, Poland; choromanska100@gmail.com (M.C.); annak04@poczta.onet.pl (A.K.); p.kosta@wp.pl (P.K.-S.); azalewska426@gmail.com (A.Z.); 2 Department of Psychiatry, Medical University of Bialystok, 16-070 Choroszcz, Poland; karolinawilczynska88@gmail.com (K.W.); hejjj@wp.pl (M.K.); nataliawygnal@gmail.com (N.O.); napoleonwas@yahoo.com (N.W.); 3 Department of Physiology, Medical University of Bialystok, 15-222 Bialystok, Poland

**Keywords:** dementia, oxidative stress, oxidative damage, saliva

## Abstract

Oxidative stress plays a crucial role in dementia pathogenesis; however, its impact on salivary secretion and salivary qualities is still unknown. This study included 80 patients with moderate dementia and 80 healthy age- and sex-matched individuals. Salivary flow, antioxidants (salivary peroxidase, catalase, superoxide dismutase, uric acid and total antioxidant capacity), and oxidative damage products (advanced oxidation protein products, advanced glycation end products (AGE), 8-isoprostanes, 8-hydroxy-2’-deoxyguanosine and total oxidant status) were estimated in non-stimulated and stimulated saliva, as well as in plasma and erythrocytes. We show that in dementia patients the concentration/activity of major salivary antioxidants changes, and the level of oxidative damage to DNA, proteins and lipids is increased compared to healthy controls. Non-stimulated and stimulated salivary secretions were significantly reduced in dementia patients. The deterioration in mini mental state examination (MMSE) score correlated with salivary AGE levels, which when considered with receiver operating characteristic (ROC) analysis, suggests their potential role in the non-invasive diagnosis of dementia. In conclusion, dementia is associated with disturbed salivary redox homeostasis and impaired secretory function of the salivary glands. Salivary AGE may be useful in the diagnosis of dementia.

## 1. Introduction

Various types of dementia are increasingly common health problems, both in developed and developing countries. It is estimated that in 2016 nearly 50 million people worldwide suffered from dementia, most of whom were over 65 years old [1]. Dementia is a group of neurodegenerative diseases characterized by progressive cognitive impairment and behavioural changes [2,3]. According to the pathophysiology, there are five basic types of dementia: Alzheimer’s dementia (AD), vascular dementia (VaD), dementia with Lewy bodies (DLB), frontotemporal dementia (FTD) and mixed dementias (MxD) [4]. Over 50% of all dementia instances are AD [5] in which amyloid β and tau protein accumulate in the central nervous system (CNS). In the pathogenesis of dementia, a particularly important role is played by oxidative stress [6], which is defined as the imbalance between the production of reactive oxygen species (ROS) and the efficiency of enzymatic (e.g., salivary peroxidase; Px, glutathione peroxidase; GPx, catalase; CAT and superoxide dismutase; SOD), as well as non-enzymatic antioxidants (e.g., uric acid; UA). Oxidative stress leads to damage of cell components by oxidation [7,8] which can be observed as an increase of oxidative-modified proteins (advanced oxidation protein products; AOPP and advanced glycation end products; AGEs), lipids (8-isoprostanes; 8-isop) and nucleic acids (8-hydroxy-2′-deoxyguanosine; 8-OHdG).

Oxidative stress is believed to be responsible for neurodegenerative diseases, causing mitochondrial dysfunction, exacerbation of inflammatory states, disorder of expression and phosphorylation of proteins involved in antioxidant protection and accumulation of neurotoxic proteins. It has been demonstrated that deposition of amyloid β and tau protein in the course of dementia is associated with excessive production of ROS, leading to oxidative damage to DNA, proteins and lipids, not only within the CNS but also in skin, skeletal muscles or exocrine glands [9,10]. Accumulation of amyloid β in the secretory epithelium of salivary glands in patients with dementia most likely disrupts the local redox balance and is responsible for impairment of the structure and function of salivary glands [11]. It has been proven that changes in the quantitative and qualitative composition of saliva in dementia patients may entail significant deterioration in the quality of life of patients, as these changes cause problems with swallowing, impair digestion and cause inflammatory and fungal lesions in the oral cavity [11,12,13]. It is very likely that oxidative stress is a key factor causing dysfunction of salivary glands in patients with dementia, in a way that is similar to what is observed in metabolic (insulin resistance [14], obesity [15] and diabetes [16,17]) and autoimmune diseases (Sjögren syndrome and rheumatoid arthritis [18]). However, little is known about the role of oxidative stress in the process of damaging salivary glands in dementia patients, as well as the use of salivary oxidative stress markers in the diagnosis of various types of dementia. It is believed that saliva could be used as an alternative diagnostic material to blood plasma or serum for the determination of redox homeostasis biomarkers. Therefore, the aim of our work was to evaluate both the secretory function of salivary glands and enzymatic and non-enzymatic antioxidant defences, in addition to oxidative damage of lipids, proteins and DNA in non-stimulated (NWS) and stimulated (SWS) saliva, as well as plasma and erythrocytes of dementia patients compared to healthy subjects.

## 2. Results

### 2.1. Clinical Findings

The study included 80 patients with moderate dementia and 80 healthy people age- and sex-matched to the study group. Dementia patients showed significantly lower mini mental state examination (MMSE) scores than the control group. There was no significant relationship between age and sex of patients with clinical and demographic parameters. The detailed patient characteristics are summarized in Table 1.

### 2.2. Dental Examination 

Decayed, missing, filled teeth (DMFT), papilla bleeding index (PBI), gingival index(GI) and the occurrence of carious lesions of root cement (CR) were used to assess the condition of oral cavity health. We found no statistical differences in DMFT, PBI, GI and CR between patients with dementia and healthy controls (Table 2).

### 2.3. Salivary Flow, Total Protein and pH of Saliva

**Figure 1:**
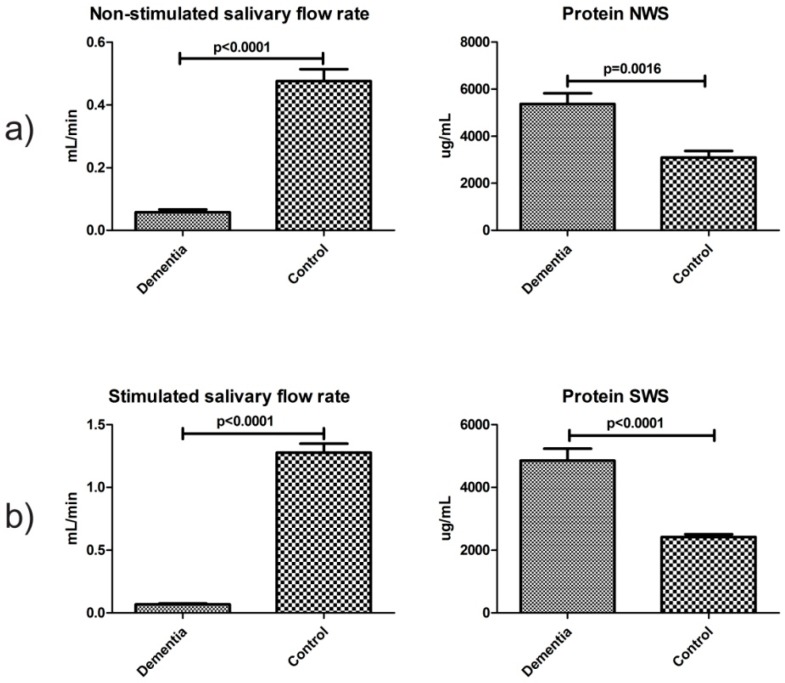
Salivary flow and total protein in non-stimulated (**a**) and stimulated (**b**) saliva of dementia patients and control group. Abbreviations: NWS, non-stimulated whole saliva; SWS, stimulated whole saliva.

For evaluation of secretory function of salivary glands, non-stimulated and citric acid-stimulated salivary secretion and total protein content in saliva were measured. The mean value of non-stimulated and stimulated salivary flow in the dementia group was significantly lower compared to the control group (*p* ˂ 0.001 and *p* ˂ 0.001, respectively). In both non-stimulated and stimulated saliva, the mean total protein concentration of dementia patients was considerably higher than that in the control group (*p* ˂ 0.01 and *p* ˂ 0.001, respectively) (Figure 1). The mean pH of non-stimulated and stimulated saliva in dementia patients was significantly lower in comparison to the control group (*p* ˂ 0.002 and *p* ˂ 0.003, respectively) (Table 3).

### 2.4. Non-Enzymatic and Enzymatic Antioxidants

**Figure 2:**
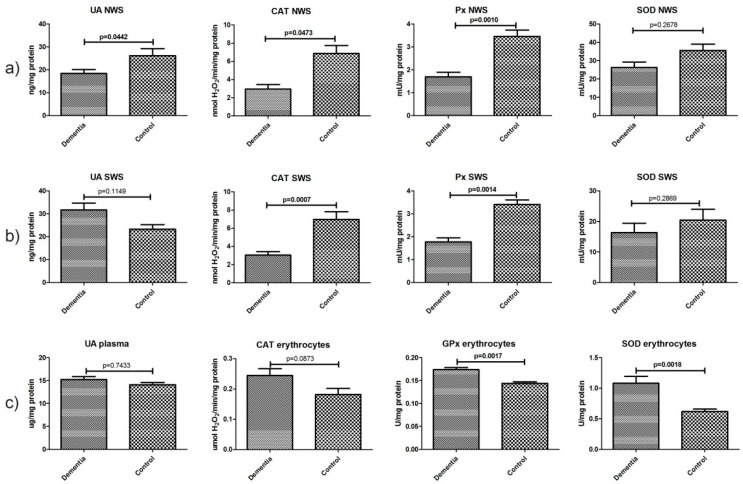
Non-enzymatic and enzymatic antioxidants in non-stimulated (**a**) and stimulated (**b**) saliva as well as plasma/erythrocytes (**c**) of dementia patients and the control group. Abbreviations: CAT, catalase; NWS, non-stimulated whole saliva; Px, salivary peroxidase; GPx, glutathione peroxidase; SOD, superoxide dismutase-1; SWS, stimulated whole saliva, UA, uric acid.

The mean concentration of non-enzymatic uric acid (UA) in the NWS of dementia patients was significantly lower compared to the control group (*p* < 0.05). CAT and Px activities were considerably lower in non-stimulated saliva compared to the controls (*p* ˂ 0.05 and *p* ˂ 0.002, respectively), as well as in stimulated saliva (*p* ˂ 0.008 and *p* ˂ 0.002, respectively). Erythrocyte GPx and SOD showed statistically higher activity in patients with dementia compared to the healthy control (*p* ˂ 0.002 and *p* ˂ 0.0019, respectively) (Figure 2).

### 2.5. Total Antioxidant/Oxidant Status

**Figure 3:**
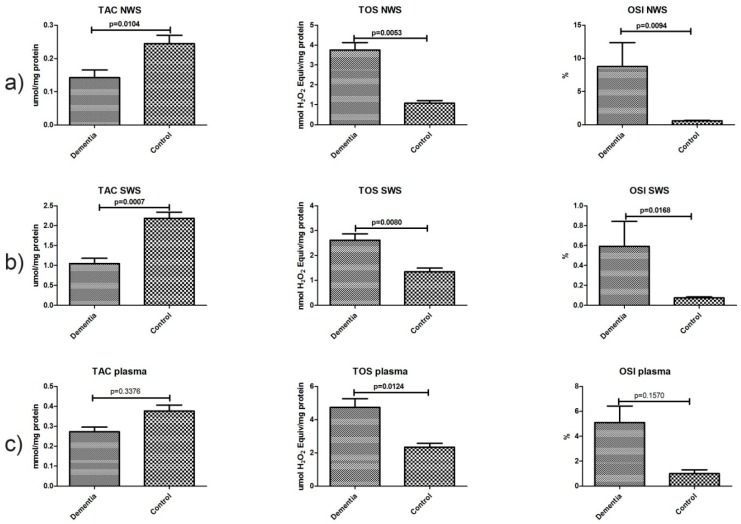
Total antioxidant/oxidant status in non-stimulated (**a**) and stimulated (**b**) saliva as well as plasma (**c**) of dementia patients and the control group. Abbreviations: NWS, non-stimulated whole saliva; OSI, oxidative stress index; SWS, stimulated whole saliva; TAC, total antioxidant capacity; TOS, total oxidant status.

In non-stimulated saliva, mean total oxidant status (TOS) and oxidative stress index (OSI) values in the dementia group were significantly higher than those in the control group (*p* ˂ 0.006 and *p* ˂ 0.01, respectively). TOS and OSI were considerably higher in the stimulated saliva of the dementia group compared to the control group (*p* ˂ 0.009 and *p* ˂ 0.02, respectively). The mean total antioxidant capacity (TAC) values in NWS and SWS in the dementia group were much lower than those in the control group (*p* ˂ 0.02 and *p* ˂ 0.001, respectively). In the plasma of dementia patients, only the TOS value was significantly higher compared to that in healthy controls (*p* ˂ 0.02) (Figure 3).

### 2.6. Oxidative Damage Products

**Figure 4:**
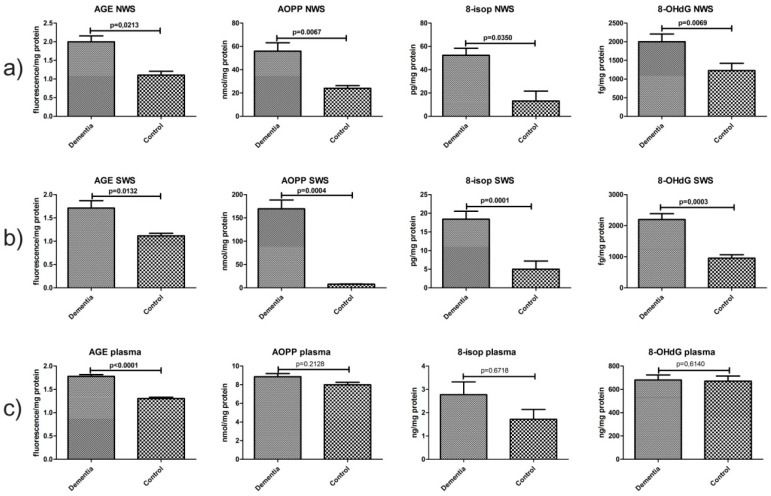
Oxidative damage to proteins, lipids and DNA in non-stimulated (**a**) and stimulated (**b**) saliva as well as plasma (**c**) of dementia patients and the control group. Abbreviations: 8-isop, 8-isoprostanes; 8-OHdG, 8-hydroxy-2′-deoxyguanosine; AGE, advanced glycation end products; AOPP, advanced oxidation protein products; NWS, non-stimulated whole saliva; SWS, stimulated whole saliva.

The mean fluorescence of AGE, mean concentrations of AOPP, 8-isop and 8-OHdG in non-stimulated saliva of dementia patients were significantly higher than those of the controls (*p* ˂ 0.03, *p* ˂ 0.007, *p* ˂ 0.04 and *p* ˂ 0.007, respectively). Mean fluorescence of AGE, mean concentrations of AOPP, 8-isop and 8-OHdG in stimulated saliva in the dementia group were considerably higher than those in the control group (*p* ˂ 0.02, *p* ˂ 0.02, *p* ˂ 0.001 and *p* ˂ 0.0004, respectively). In the plasma of dementia patients, only the mean value of AGE was significantly higher than that in healthy controls (*p* < 0.001) (Figure 4).

### 2.7. Correlations

The observations in dementia patients revealed a negative correlation between 8-isop concentration in NWS and non-stimulated salivary flow rate (*p* = 0.019, r = −0.75), as well as a negative correlation between SWS 8-isop concentration, AGE content and stimulated salivary flow rate (*p* = 0.03, r = −0.692 and *p* = 0.029, r = −0.54, respectively). A positive relationship between SWS OSI and stimulated salivary secretion (*p* = 0.01, r = 0.455) was also demonstrated. In stimulated saliva, TAC was negatively correlated (*p* = 0.008, r = −0.35), while OSI was positively correlated with total salivary protein (*p* = 0.007, r = 0.36). A positive correlation between TOS and total protein concentrations in NWS (*p* = 0.008, r = 0.47) was also observed in dementia patients. The deterioration in MMSE score was connected to high NWS levels of AGE in the study group (*p* = 0.04, r = −0.45). We did not find significant differences in the concentration/activity of the tested antioxidants and products of oxidative stress between different types of dementia in NWS, SWS, plasma and erythrocytes. There was also no significant relationship between age and sex of patients and redox/oxidative stress biomarkers.

### 2.8. ROC Analysis

**Figure 5:**
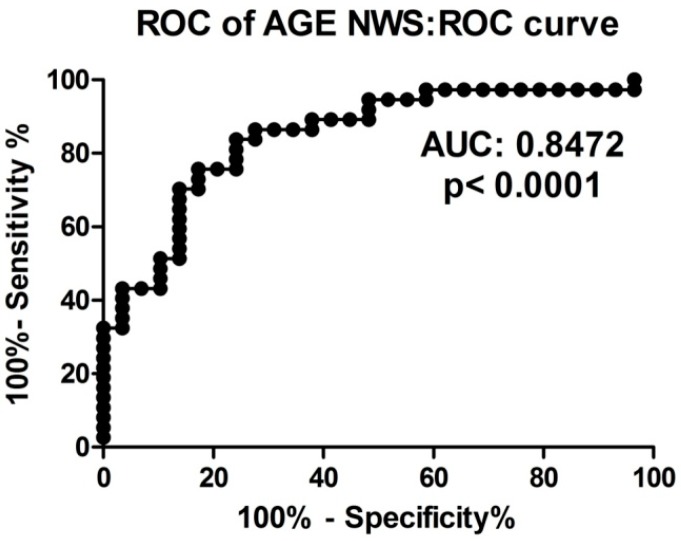
ROC analysis of AGE content in non-stimulated saliva of dementia patients compared to healthy controls. Abbreviations: AUC, area under curve; NWS, non-stimulated whole saliva; ROC, receiver operating characteristic.

In this study, we performed the receiver operating characteristic (ROC) analysis of discriminative abilities of selected oxidative stress biomarkers in the diagnosis of dementia patients. The usefulness of salivary redox parameters and oxidative damage products in dementia patients are presented in Table 4. Particular attention should be paid to NWS TOS, NWS and SWS OSI, SWS AOPP, NWS and SWS 8-isop, as well as SWS 8-OHdG, whose AUC is close to or equal to 1.0 between dementia patients and the control group (Table 4). We also showed a very high diagnostic value of AGE determination in non-stimulated saliva of dementia patients compared to healthy controls. The optimal AGE fluorescence (AUC 0.85, *p* < 0.0001) in the NWS, differentiating the group of dementia patients from the control group, was the value <1.451 fluorescence/mg protein with sensitivity of 75.68% and specificity of 75.86% (Table 4, Figure 5).

## 3. Discussion

This is the first study to evaluate the oxidant/antioxidant status and oxidative stress markers in non-stimulated and stimulated saliva, as well as in plasma/erythrocytes of the elderly with different types of dementia. Firstly, we have proven that dementia patients reveal decreased antioxidant properties of saliva and increased levels of the products of DNA, protein and lipid oxidative damage, with simultaneous reduced secretion of non-stimulated and stimulated saliva. Secondly, our results suggest that changes in salivary redox homeostasis are independent of systemic changes in the course of dementia (plasma/erythrocytes). However, the assessment of salivary AGE could be one of the non-invasive biomarkers in diagnosing dementia.

Aging is a complex biological process that leads to gradual tissue and organ failure [19]. Physical decline in aging also impairs mental health and influences psychosocial relationships, which renders independent life impossible and deepens alienation [20]. In addition, the aging process significantly affects the health of the human oral cavity. Elderly patients suffer from atrophic changes in the parotid, submandibular and sublingual glands. The parenchyma of salivary glands is replaced by fibrous connective and adipose tissue, and the number of serous vesicles is reduced [21,22]. These changes result in salivary gland hypofunction. Although the exact cause of salivary gland abnormalities is still not well known, it is believed that oral pathologies associated with dementia, as well as other dementia-associated diseases, are mainly caused by oxidative stress and disturbances in redox homeostasis.

Enzymatic and non-enzymatic antioxidants play an important role in counteracting oxidative stress. These compounds prevent the formation of ROS, and thus inhibit their interactions with cellular components and stop free radical reactions. In this study, both the activity of antioxidant enzymes (Px and CAT) as well as UA and TAC concentrations were significantly lower in NWS and SWS of patients with dementia. It can be assumed that the decrease in activity of salivary antioxidants may result from the modification of enzyme molecules caused, directly or indirectly, by ROS activity, whereas the decrease of salivary UA may be caused, directly or indirectly, by the depletion of salivary antioxidant reserves in the course of dementia. It appears that reducing the antioxidant properties of saliva may indicate an increased susceptibility of the salivary glands to oxidative damage, and dramatically increase the risk for development of oxidative stress-related oral maladies (dental caries, burning mouth syndrome and oral inflammatory infections like gingivitis, periodontitis, oral mucosa ulceration and candidiasis) [23]. On the other hand, the increase in GPx and SOD activity in erythrocytes suggests a central adaptive response of the body to excessive production of ROS in these patients, so changes in redox balance within salivary glands are different from systemic lesions.

One of the most important sources of ROS in patients with dementia is amyloid β, which in addition to the brain, is also deposited in peripheral regions (nasal mucosa, lacrimal or lingual glands) including cells of salivary gland epithelium [11]. It has been demonstrated that amyloid β forms oligomers that can directly increase the formation of hydrogen peroxide (showing SOD-like enzymatic activity), activate NADPH oxidase (nicotinamide adenine dinucleotide phosphate-oxidase), which is the primary source of free radicals in the cell, and increase ROS production in mitochondria by modulating the activity of alcohol dehydrogenase that binds amyloid β and α-ketoglutarate dehydrogenase [24]. The effect of free radical activity is the disturbance in the structure and function of cellular biomolecules, which may lead to their total dysfunction and, ultimately, cell death. It has been shown that patients with dementia have intracellular oxidative damage in the brain, heart, liver, kidneys and lungs [9,25]. These include, inter alia, lipid peroxidation of cell membranes, inactivation of enzymes, protein aggregation and damage of nucleic acids, and these processes aggravate with age [26,27].

Via our experiments, we were the first to notice the increase of oxidative damage to proteins (↑AGE, ↑AOPP), lipids (↑8-isop) and DNA (↑8-OHdG) in both NWS and SWS of dementia patients compared to elderly people without such disorders. It is assumed that damage to cellular components by oxidation may lead to impairment of the structure and function of salivary glands as well as affect the composition of the secreted saliva [22,23]. Such changes have been observed in salivary gland dysfunction in other disease syndromes with proven influence of oxidative stress (e.g., diabetes, Sjögren’s Syndrome and rheumatoid arthritis) as well as in aging [22,28,29,30]. The lack of correlation between parameters of oxidative stress in saliva and plasma indicates that disturbances in the course of dementia are of different nature in salivary glands (saliva) and throughout the body (blood).

The results of our study also indicate a significant impairment of the secretion activity of salivary glands in patients with dementia, which results in decreased non-stimulated and stimulated salivary flow. The possible influence of oxidative stress on hypofunction of the submandibular glands is expressed as a negative correlation between 8-isop concentration and NWS flow, and the influence on the parotid glands is expressed as a negative correlation between 8-isop concentration, AGE content and SWS flow, as well as a positive correlation between OSI and stimulated salivary secretion. It should be noted that in the absence of stimulation, the submandibular glands provide about 60% of the volume of mixed saliva, thus forming the main source of non-stimulated saliva, while upon stimulation, only the parotid glands increase their secretion, whereby the latter can be considered as the main source of stimulated saliva [31]. Another parameter used to evaluate salivary gland function is total protein concentration [15,32]. In this study, the increase in salivary protein in dementia patients most likely results from a decrease in the total volume of secreted saliva [33]. It appears that correlations between oxidative damage markers and salivary protein may indicate the influence of oxidative stress on the reduction of protein secretion into saliva in these group of elderly patients. It can also be assumed that the decrease in saliva secretion in patients with dementia may be of a central nature and, like Alzheimer’s disease, is caused by the partial loss of neurons within the CNS. Structural abnormalities within the pons, from which parasympathetic fibres responsible for the secretion of the submandibular glands are derived, have been demonstrated in patients with Alzheimer’s disease [34]. We also cannot exclude the effects of pharmacotherapy on salivary secretion, as well as salivary composition, in patients with dementia, although the number of subjects taking drugs was similar in the control and the study group. It is estimated that over 500 medications may lead to hyposalivation and over 80% of all medicines cause dry mouth (xerostomia) [35]. In our study, we did not observe significant differences in salivary flow and composition between patients receiving and those not receiving the drugs, which is probably due to the small size of the group not taking any medication.

Recently, the use of oxidative stress biomarkers in the diagnosis of various oral and systemic diseases has been highlighted. Saliva seems to be a particularly interesting diagnostic material, as it is obtained easily and non-invasively, which significantly reduces the discomfort associated with blood sampling [36]. In this study, we have shown very high diagnostic value of determination of salivary AGE in the diagnosis of dementia in elderly people compared to those without dementia disorders. The content of AGE in NWS revealed significant negative correlation with the MMSE result, and hence, salivary AGE could be used in the diagnosis of dementia. It is also worth reminding that the diagnostic utility of AGE has been demonstrated in the diagnosis and monitoring of the progression of Alzheimer’s disease and diabetes, as well as renal, cardiovascular and pulmonary diseases [2,37]. Our study, however, included only patients with moderate dementia, so that it is still unknown whether changes in salivary AGE could reflect clinical progression of the disease. On the other hand, high AUC values of salivary oxidative stress products (AOPP, 8-isop and 8-OHdG) as well as NWS TOS, and NWS and SWS OSI may indicate the usefulness of these parameters to evaluate salivary gland dysfunction in dementia patients. However, further research and clinical observations of elderly patients with dementia are needed.

Analysing the results of our experiments, its limitations and imperfections should also be considered. We evaluated only selected markers of redox homeostasis and oxidative damage, so the evaluation of other parameters may yield different results and lead to different conclusions. Patients with hypertension and coronary heart disease were included in both the study group and in the control group, so the influence of these disorders and their drug treatments on the analysed oxidative stress markers and salivary secretion activity could not be completely eliminated. Undoubted advantages of the experiment are: the relatively high number of participants selected carefully based on their accompanying illnesses and medications taken, as well as the fact that it is the first study to compare enzymatic and non-enzymatic antioxidant systems and oxidative damage to saliva and plasma/erythrocytes in dementia patients. It should also be remembered that the observed disorders of antioxidant systems and the increased formation of oxidative stress products do not necessarily result from the aforementioned pathological changes in the course of dementia, but may be a consequence of this disease.

## 4. Materials and Methods

The research was approved by the bioethics Committee of the Medical University of Bialystok, Poland (permission number R-I-002/62/2016). After a thorough explanation of the purpose of the study and its possible risks, all participants of the study consented in writing to participate in the experiment.

### 4.1. Patients

The study group consisted of 80 dementia patients (55 women and 25 men) treated from November 2016 to March 2017 at the Psychogeriatrics Department of the Dr. Stanisław Deresz Independent Public Psychiatric Health Care Centre in Choroszcz. Based on psychiatric, psychological and additional examinations, the group was divided into three subgroups: patients with Alzheimer’s dementia (24 patients), with vascular dementia (30 patients) and with mixed dementia (26 patients). The study material was collected from all patients in the study group prior to the commencement of pharmacological and psychological treatment.

All patients were examined by the same experienced psychiatrist (N.W.). The criteria for inclusion in the study group covered: cognitive impairment with undisturbed consciousness seen in the clinical picture and confirmed by the mini mental state examination (MMSE) indicating a moderate dementia (score between 11 and 18 points on a 30-point scale), at least 6 months of positive history of cognitive impairment, and no history of psychoactive substance abuse. Moreover, head CT scans of the patients excluded instances of acute haemorrhage and ischemia, tumours of the central nervous system and normal pressure hydrocephalus.

The control group, selected by sex and age to match the study group, consisted of 80 patients attending follow-up visits at the Department of Restorative Dentistry at the Medical University of Bialystok from November 2016 to March 2017. The experiment included people whose scores in the MMSE examination were higher than 23 points.

Only patients with normal results of complete blood count (erythrocytes, leukocytes, haemoglobin, platelets and hematocrit) and biochemical blood tests (sodium, potassium, creatinine, ASPAT, ALAT, International Normalized Ratio; INR and CRP), as well as normal levels of TSH, calcium, vitamin B_12_ and folic acid were admitted to the study and control groups. The exclusion criterion in both groups was any chronic systemic or autoimmune disease (diabetes, rheumatoid arthritis and psoriasis) and also lung, thyroid, liver, kidney, gastrointestinal and infectious diseases (HCV and HIV infection) and immunity disorders. Additionally, smokers and patients taking antibiotics, non-steroidal anti-inflammatory drugs, glucocorticosteroids, vitamins and dietary supplements were excluded from the study.

### 4.2. Blood Collection

Venous blood (10 mL) was collected from all patients on empty stomach, upon overnight rest, using S-Monovette^®^ K3 EDTA blood collection system (Sarstedt, Germany). Immediately after collection, blood was centrifuged at 1500× *g* for 10 min at +4 °C (MPW 351, MPW Med. Instruments, Warsaw, Poland) to separate plasma from erythrocytes. The top layer (plasma) was taken, and the erythrocytes were washed three times in cold saline solution (0.9% *w*/*v*). Then erythrocytes were haemolysed by adding 9 volumes of cold 50 mM phosphate buffer of pH 7.4 (*v*:*v*) [38]. Samples were protected from exposure to light. In order to provide protection against oxidation processes, butylated hydroxytoluene antioxidant (BHT, Sigma-Aldrich, Saint Louis, MO, USA; 5 μL 0.5 M BHT in acetonitrile per 0.5 mL plasma/erythrocytes) was added to the obtained supernatants [14]. The samples were frozen at the temperature of −80 °C in which they were stored until being assayed.

### 4.3. Saliva Collection

The study material was whole saliva, both non-stimulated (NWS) and stimulated (SWS), collected from patients via the spitting method. The subjects had not consumed food or beverages other than pure water, and had refrained from oral hygiene activities for at least 2 h prior to saliva collection. Moreover, the patients in the study/control group had not taken any medicines for at least 8 h before saliva collection.

Saliva was always collected in the same circumstances: between 8 a.m. and 10 a.m. (to minimize the effect of daily rhythm on salivation) in the same, separate and quiet room so that the patients did not feel uncomfortable or upset. After at least 5 min of adaptation to the room environment and upon rinsing the mouth with distilled water at room temperature three times, the patients had their saliva collected in a seated position, with the head slightly inclined downwards, trying to refrain from facial and lip movements. The saliva gathered at the bottom of the oral cavity was spat into a sterile centrifuge tube placed in a container with ice. Saliva collected in the first minute was discarded. The NWS collection time was 10 min [15]. After a 5 min break, the collection of SWS begun. Salivation was stimulated by sprinkling the tongue every 30 s with 10 μL of 2% citric acid. SWS was collected for 5 min to a maximum volume of 5 mL, in the same manner as the NWS [15]. The volume of each saliva sample was measured with a pipette calibrated to 100 μL. The minute flow of NWS and SWS was calculated by dividing the saliva volume by the time necessary for its secretion. Upon collection, saliva was immediately centrifuged (20 min, 3000× *g*, +4 °C; MPW 351, MPW Med. Instruments, Warsaw, Poland). In order to protect the obtained supernatants against oxidation processes, butylated hydroxytoluene (BHT, Sigma-Aldrich, Sigma-Aldrich, Saint Louis, MO, USA; 5 μL 0.5 M BHT in acetonitriles per 0.5 mL saline fluid) was added and the supernatants were frozen at −80 °C. The samples were stored at that temperature until the performance of an assay [14]. Saliva samples for pH determination were analysed immediately after collection with the SevenMulti Mettler Toledo pH meter (Mettler-Toledo, Columbus, OH, USA).

### 4.4. Dental Examination

Dental examinations were performed in artificial lighting, using a mirror, an explorer and a periodontal probe according to the criteria of the World Health Organization [39]. The same dentist (M.C.) performed every examination after the collection of non-stimulated and stimulated saliva. The dental examination included measurement of decayed, missing, filled teeth (DMFT), papilla bleeding index (PBI), gingival index (GI) and the occurrence of carious lesions of root cement (CR). The DMFT index is the sum of the teeth with caries (D), teeth extracted because of caries (M), and teeth filled due to the occurrence of caries (F). The PBI showed the intensity of bleeding from the gingival papilla after probing [40]. The GI criteria included qualitative changes in the gingival [41]. In 30 patients, the inter-rater agreements between the examiner (M.C.) and two other experienced dentists (A.Z. and A.K.) were assessed. The reliability for DMFT was r = 0.96; for PBI: r = 0.98; for GI: r = 0.98; and for CR = 0.96. Cohen kappa (online calculator) was used to establish the inter-rater agreement between the two examiners.

### 4.5. Biochemical Analysis

The performed analysis included: salivary flow, pH, total protein, antioxidant enzymes (salivary peroxidase; Px (C 1.11.1.7), glutathione peroxidase; GPx (EC 1.11.1.9), catalase; CAT (EC 1.11.1.6), Cu-Zn superoxide dismutase; SOD-1 (E.C. 1.15.1.1)), non-enzymatic antioxidants (uric acid; UA, total antioxidant capacity; TAC), as well as oxidative damage products (advanced oxidation protein products; AOPP, advanced glycation end products; AGEs, 8-isoprostanes; 8-isop, 8-hydroxy-2′-deoxyguanosine; 8-OHdG, and total oxidant status; TOS).

### 4.6. Salivary, Plasma and Erythrocytes Antioxidants

CAT, SOD-1 and total protein were estimated in NWS, SWS and erythrocytes, while UA, TAC and total protein were analysed in NWS, SWS and plasma. Px was evaluated only in NWS and SWS, while GPx was only evaluated in erythrocytes. All assays were performed in duplicate, except for CAT and TAC determination (see below). The results were standardized to mg of total protein. The absorbance in all assays was measured using Infinite M200 PRO Multimode Microplate Reader, Tecan (Tecan Group Ltd., Männedorf, Switzerland).

Px activity was determined by a colorimetric method according to Mansson-Rahemtulla et al. [42]. This method is based on the reduction of 5,5′-dithiobis-(2-nitrobenzoic acid) (DTNB) to thionitrobenzene acid that reacts with OSCN (hypothiocyanite; product of KSCN (potassium thiocyanate) oxidation by Px). A decrease in the concentration of thionitrobenzene acid depending on the activity of Px was measured five times at 412 nm wavelength at 30 sec intervals.

GPx activity was measured by the colorimetric method described by Paglia and Valentine [43]. The basis of this method is oxidation of reduced glutathione (GSH) and reduction of organic peroxides by GPx, associated with regeneration of oxidized glutathione (GSSG) in the presence of NADPH and glutathione reductase (EC 1.6.4.2). One unit of GPx activity (1 unit) was assumed to catalyze oxidation of 1 μmol of NADPH for 1 min (25 °C and pH 7.4).

CAT activity was determined colorimetrically according to the Aebi method [44], by measuring the decomposition rate of hydrogen peroxide (H_2_O_2_) in phosphate buffer at pH 7.0 and 240 nm wavelength. One unit of CAT activity was defined as the amount of enzyme that decomposes 1 mmol H_2_O_2_ for 1 min. CAT activity was determined in triplicate samples.

SOD-1 activity was determined by the colorimetric method described by Misra and Fridovich [45], measuring the cytosolic activity of SOD by inhibiting the oxidation of epinephrine to adrenochrome at pH 10.2 and 26 °C. It was assumed that one unit of SOD-1 activity inhibits the oxidation of epinephrine by 50%.

UA concentrations were measured colorimetrically with the commercial QuantiChrom^TM^ Uric Acid DIUA-250 kit (BioAssay Systems, Harward, CA, USA), as instructed by the manufacturer. In this method, in the presence of UA, 2,4,6-tripyridyl-s-triazine forms a blue complex with iron ions, the intensity of which at 630 nm wavelength is directly proportional to the UA content of the sample.

TAC concentration was measured by the colorimetric method described by Erel [46], using 2,2-azinobis-3-ethylbenzothiazoline-6-sulfonic acid radical cation (ABTS*^+^). Changes in the absorbance of the ABTS*^+^ solution caused by the antioxidants contained in the sample, and their antioxidant properties were measured at 660 nm. TAC was calculated from the calibration curve for the vitamin E analogue Trolox (6-hydroxy-2,5,7,8-tetramethylchroman-2-carboxylic acid). TAC content determination was performed in triplicate.

Total protein concentration was determined by a colorimetric method with the commercial Thermo Scientific PIERCE BCA Protein Assay kit (Rockford, IL, USA), using the BCA method [47]. Bicinchoninic acid (BCA) and copper ions form a stable complex which has a maximum absorption at λ of 562 nm.

### 4.7. Salivary and Plasma Oxidative Modification Products

Oxidative modification products (AOPP, AGE, 8-isop and 8-OHdG) as well as TOS and total protein were analysed in the NWS, SWS and plasma. All assays were performed in duplicate, except for TOS determination (see below). The absorbance/fluorescence in all assays was measured using Infinite M200 PRO Multimode Microplate Reader, Tecan.

AOPP concentration was determined by the colorimetric method described by Kalousová et al. [48], by measuring the oxidative capacity of the iodine ion at 340 nm wavelength. For the determination of AOPP concentration in plasma, plasma samples were diluted in phosphate-buffered saline (PBS, pH 7.2) 1:5 (*v*:*v*). The calibration curve was prepared for chloramine solutions in the concentration range 0–100 μmol/L.

The content of AGE was determined fluorimetrically by measuring AGE-specific fluorescence at 350 nm excitation wavelength and 440 nm emission wavelength [48]. For AGE determination, plasma samples were diluted 1:5 (*v*:*v*) in PBS (pH 7.2).

Concentration of 8-isop was determined with an ELISA test using a complete set of reagents (8-Isoprostane ELISA Kit, Cayman Chemicals, Ann Arbor, MI, USA). The principle of this method is based on competition between 8-isop and 8-isop conjugate with acetylcholinesterase (Tracer) for a site for binding the antibody specifically directed against 8-isop. The ability to bind an 8-isop conjugate to acetylcholinesterase and to the aforementioned antibody is inversely proportional to the 8-isop concentration in the tested sample.

Concentration 8-OHdG was determined with an ELISA test using a commercial kit from USCN Life Science, Wuhan, China in accordance with the manufacturer’s instructions. The specific fragment of the 8-OhdG protein chain is bound by the first monoclonal antibody that covers the walls of the microplate. Then the second biotin-labelled monoclonal antibody is added. After the addition of streptravidin-conjugated horseradish peroxidase (HRP), HRP binds to biotin, and horseradish peroxidase triggers a colour reaction with o-phenylenediamine (OPD). The intensity of coloration measured at 450 nm wavelength is proportional to the concentration of 8-OHdG in the tested sample.

TOS concentration was determined bichromatically (560/800 nm) in triplicate samples according to the method described by Erel [49]. This method is based on the oxidation of Fe^2+^ to Fe^3+^ ions in the presence of the oxidants contained in the sample, and Fe^3+^ measurement with xylenol orange. The results are expressed as micromolar hydrogen peroxide equivalent per litre (μmol H_2_O_2_ Equiv./L).

Oxidative stress index (OSI) was calculated by dividing TOS by TAC, and expressed in % [28].

### 4.8. Statistical Analysis

Data was expressed as mean values ± SEM. Statistical analysis was performed using the Statistica 10.0 system (Statsoft, Cracow, Poland) according to Student’s *t*-test. Relations between various parameters were tested by Pearson’s correlation. Statistical significance was established at *p* ≤ 0.05. Due to the lack of significant differences between the different types of dementia, as well as age and sex of the subjects, results of biochemical determinations were presented as dementia (all subgroups together) and the control group. The diagnostic value and optimal cutoff levels of oxidative stress biomarkers were determined based on the analysis of surface area under the ROC curve known as the area under curve (AUC).

## Figures and Tables

**Table 1:** Demographic data and general health of the patients.

Characteristic		Alzheimer’s Dementia (AD)	Vascular Dementia (VaD)	Mixed Dementia (MxD)	All Patients with Dementia	Control Group
Sex	Male *n*	10	8	7	25	25
	Female *n*	14	22	19	55	55
Age mean (SEM)		77.87 (1.427)	80.63 (1.107)	81.81 (1.325)	80.12 (0.7455)	80.12 (0.7455)
Education in years mean (SEM)		7.636 (0.8337)	7.48 (0.5806)	5.6 (0.7158)	7.081 (0.4223)	7.52 (0.315)
MMSE score mean (SEM)		13.14 (0.5516) *	13.31 (0.5089) *	14 (0.6211) *	13.45 (0.3201) *	27.42 (0.3465) *
Hypertension *n*		8	11	18	37	34
Coronary heart disease *n*		4	5	6	15	14
Atherosclerosis *n*		3	3	2	8	9
Osteoporosis *n*		1	1	0	2	3
Polypharmacy	No drugs *n*	4	1	8	13	12
	Non-polypharmacy (1–5 drugs) *n*	14	18	13	45	43
	Polypharmacy (>5 drugs) *n*	6	11	5	22	20

Abbreviations: AD, Alzheimer’s dementia; MxD, mixed dementia; *n*, number of patients; SEM, standard error of the mean; MMSE, mini mental state examination; VaD, vascular dementia; * *p* < 0.05.

**Table 2:** Stomatological characteristics of the patients.

Characteristic	Alzheimer’s Dementia (AD) *n* = 24	Vascular Dementia (VaD) *n* = 30	Mixed Dementia (MxD) *n* = 26	All Patients with Dementia *n* = 80	Control Group *n* = 80
DMFT mean (SEM)	28.00	30.09	29.96	29.50	29.32
	(1.62)	(0.6885)	(0.8902)	(0.5907)	(0.5295)
PBI mean (SEM)	1.554	1.973	1.520	1.763	1.087
	(0.341)	(0.239)	(0.2687)	(0.1619)	(0.06926)
GI mean (SEM)	1.750	2.175	2.063	2.026	1.582
	(0.3493)	(0.1377)	(0.1475)	(0.1191)	(0.06122)
CR mean (SEM)	0.1875	0.5957	0.1304	0.3248	0.21
	(0.1008)	(0.3765)	(0.09544)	(0.1827)	(0.128)

Abbreviations: AD, Alzheimer’s dementia; CR, root caries; DMFT, decayed, missing, filled teeth index; GI, gingival index; MxD, mixed dementia; *n*, number of patients; PBI, papilla bleeding index; SEM, standard error of the mean; VaD, vascular dementia.

**Table 3:** Salivary pH.

Parameter	Dementia *n* = 80	Control Group *n* = 80
pH NWS	7.038	7.649
	(0.09722)	(0.05961) *
pH SWS	6.672	7.328
	(0.1285)	(0.1007) *

Abbreviations: NWS, non-stimulated whole saliva; SWS, stimulated whole saliva; *n*, number of patients; * *p* < 0.005.

**Table 4:** Receiver operating characteristic (ROC) analysis of salivary markers of oxidative stress in dementia patients and healthy controls.

Parameter	AUC		Cutt-Off		Sensitivity		Specificity	
	NWS	SWS	NWS	SWS	NWS	SWS	NWS	SWS
*Non-enzymatic and Enzymatic Antioxidants*								
UA	0.6039 (0.07348)	0.6243 (0.07156)	>18.12	<25.45	57.58	58.06	57.58	58.06
CAT	0.7495 (0.05887)	0.7758 (0.05452)	>3.233	>3.487	64.86	70.27	63.33	67.65
Px	0.8342 (0.04984)	0.8728 (0.04459)	>2.555	>2.574	72.97	83.78	73.33	82.35
SOD	0.6343 (0.06919)	0.5686 (0.06920)	>25.00	>10.71	60	55.56	56.67	55.88
*Total Antioxidant/Oxidant Status*								
TAC	0.737 (0.06168)	0.8358 (0.05134)	>0.1586	>1.514	66.67	80.56	66.67	79.41
TOS	0.9213 (0.03193)	0.7712 (0.05522)	<1.837	<1.554	80.56	63.89	80	64.71
OSI	0.9080 (0.03687)	0.9044 (0.03488)	<1.064	<0.1259	80.56	77.78	79.31	76.47
*Oxidative Damage Products*								
AGE	0.8472 (0.04834)	0.7035 (0.06587)	<1.451	<1.258	75.68	64.86	75.86	64.71
AOPP	0.8135 (0.05092)	1 (0.0)	<31.24	<43.49	70.27	100	70	100
8-isop	0.9286 (0.06883)	0.9231 (0.05504)	<18.67	<8.236	92.86	84.62	94.12	87.5
8-OHdG	0.7559 (0.09378)	0.9899 (0.01631)	<1361	<1567	70	88.89	70.59	90.91

Abbreviations: 8-isop, 8-isoprostanes; 8-OHdG, 8-hydroxy-2′-deoxyguanosine; AUC, area under curve; AGE, advanced glycation end products; AOPP, advanced oxidation protein products; CAT, catalase; NWS, non-stimulated whole saliva; OSI, oxidative stress index; Px, salivary peroxidase; SOD, superoxide dismutase-1; SWS, stimulated whole saliva; TAC, total antioxidant capacity; TOS, total oxidant status; UA, uric acid.
